# Melatonin Improved the Survival of Multi-Territory Perforator Flaps by Promoting Angiogenesis and Inhibiting Apoptosis via the NRF2/FUNDC1 Axis

**DOI:** 10.3389/fphar.2022.921189

**Published:** 2022-05-24

**Authors:** Chengxiong Huang, Liming Qing, Xiaoyang Pang, Jinfei Fu, Yu Xiao, Juyu Tang, Panfeng Wu

**Affiliations:** Department of Orthopedics, Hand and Microsurgery, National Clinical Research Center of Geriatric Disorders, Xiangya Hospital of Central South University, Changsha, China

**Keywords:** apoptosis, angiogenesis, NRF2/FUNDC1, perforator flap, melatonin

## Abstract

**Background:** Multi-territory perforator flaps are a reconstructive measure for repairing large soft tissue defects caused by tumors or trauma. However, the use of these flaps in clinical practice has been restricted due to the uncertain blood supply. Therefore, promoting the survival of the multi-territory perforator flap is critical for clinical repair and reconstruction. In our study, we explored the effects of melatonin (MLT) on multi-territory perforator flaps and the possible molecular mechanisms.

**Materials and Methods:** Seventy-two Sprague–Dawley rats (250–300 g) were randomly divided into 3 groups (*n* = 24): Control, MLT and MLT + ML385 groups. First, we assessed the survival area of the flap, followed by the micro-vessel density and CD31-positive vessel expression. Apoptosis of the skin flap under immunofluorescence and expression of the apoptosis-related proteins Bcl-2, Bax and Caspase3 were measured. Additionally, angiogenesis of the skin flaps was shown by angiography, and NRF2 and FUNDC1 mRNA and protein expression was detected by real-time PCR and western blotting.

**Results:** The results showed that MLT increased the survival area of the multi-territory perforator flap, which was related to increased angiogenesis and decreased apoptosis. We also found that mRNA and protein of NRF2 and FUNDC1 levels were significantly increased after MLT treatment, and an NRF2 inhibitor reversed the ability of MLT to enhance multi-territory perforator flap survival, promote angiogenesis and inhibit apoptosis and reduced FUNDC1 protein expression.

**Conclusion:** MLT promoted angiogenesis and inhibited apoptosis to promote the survival of multi-territory perforator flaps, which may be regulated via the NRF2/FUNDC1 axis.

## 1 Introduction

Multi-territory perforator flaps are regarded as an effective reconstruction measure for large soft tissue defects caused by trauma or tumors (Mokdad et al., 2016; Zhang et al., 2016; Qing et al., 2017; Luo et al., 2019; Qing et al., 2019). However, the uncertainty blood supply of multi-territory perforator flaps can lead to 5.9 to 22 percent rate of flap necrosis. (Okşar et al., 2001; Chang et al., 2004; Bigdeli et al., 2020; Salibian et al., 2021), and which can result in longer hospital stays, second operation, and more treatment cost (Tang et al., 2013; Qing et al., 2015). Hence, improving the survival of multi-territory flaps has vital clinical significance. In the past, the main way to harvest a multi-territory perforator flap was through the “delay flap” technique (Callegari et al., 1992; Coşkunfirat et al., 2000; Acartürk et al., 2015). However, due to the limitation of the perforator vessel, it was difficult to harvest sufficient tissue for the traditional multi-territory perforator flap to repair large soft tissue defects. Nowadays, researcher (Okşar et al., 2001; Chang et al., 2004; Hallock and Rice 2005; Luo et al., 2021) have reported the method of arterial supercharging and venous superdrainage. Wu et al. (Wu et al., 2021)have reported a flap of distal arterialized venous supercharging, which also could alleviate distal ischemia and improve flap survival in rats. While microsurgeons need anastomose additional blood vessels and cost more time, even though, the distal portion of perforator flaps often lacks suitable caliber vessels for anastomosis. As a result, we need to explore simple and effective methods to promote the survival of multi-territory flaps. Based on the concept of the angiosome proposed by Taylor in 1986, choke vessels are bridges connecting adjacent angiosomes (Taylor and Palmer 1987). Previous studies have demonstrated that inducing choke vessels to “true anastomosis” could improve the survival of the flap area (Taylor and Palmer 1987; Schaverien et al., 2009; Taylor et al., 2013). Stalder et al. (Stadler et al., 2016) proposed that remodeling of choke vessels is closely related to the angiogenesis of multi-territory perforator flaps. Additionally, the regulation of apoptosis plays a significantly critical role in the survival areas of multi-territory perforator flaps (Lin et al., 2019; Jiang J. et al., 2021; Bali et al., 2021; Kim et al., 2021). As a result, apoptosis and angiogenesis might transform vessel remodeling, which could promote flap survival. However, the molecular mechanism by which flap survival is enhanced is unclear.

Melatonin (MLT) is one of the hormones secreted by the conarium and has antioxidant stress and anti-inflammatory effects (Reiter et al., 2014; Carrascal et al., 2018). Furthermore, MLT has neuroendocrine and immunoregulatory activity (Lochner et al., 2018). Studies have shown that MLT has a protective effect against ischemia–reperfusion injury in various organs (Yeung et al., 2015; Gou et al., 2020). Furthermore, researchers have confirmed that MLT can promote angiogenesis in gastric ulcers, skin lesions and some physiologic processes (Gurlek et al., 2004; Kerem et al., 2014; Ma Q. et al., 2020). It has been proven that MLT has a protective effect on osteoarthritis, myocardial ischemia reperfusion and Parkinson’s disease by inhibiting apoptosis signaling pathways and antioxidant stress (Fernández et al., 2015; Hosseinzadeh et al., 2016; Chang et al., 2018; Tamtaji et al., 2020). As mentioned previously, flap survival was thought to be closely related to angiogenesis and apoptosis. Since MLT has antioxidative stress properties, it has been studied to reduce avascular necrosis in random skin flaps (Gurlek et al., 2004; Kerem et al., 2014). However, researchers have not focused on the effect and molecular mechanism of MLT on multi-territory skin flaps.

Therefore, we hypothesized that MLT could improve the survival of multi-territory perforator flaps through angiogenesis and by suppressing apoptosis. Therefore, this article investigates whether MLT promotes the survival of multi-territory perforator flaps and explores the related molecular mechanism.

## 2 Materials and Methods

### 2.1 Ethics Statement

All operations and surgical procedures were performed in accordance with the guidelines of the China Council of Animal Care and the approval of the Laboratory Animal Committee of Central South University.

### 2.2 Animal Experiments

Seventy-two Sprague–Dawley rats (250–300 g) were purchased from the Department of Laboratory Animals, Central South University, and divided randomly into three groups: a control group (*n* = 24), an MLT treatment group (*n* = 24), and an MLT + ML385 ((inhibitor of nuclear factor E2-related factor 2 (NRF2)) cotreatment group (*n* = 24). The rats were housed in the Department of Laboratory Animals, Central South University (Changsha, Hunan, China) and provided free access to food and water.

### 2.3 Flap Animal Model

A multi-territory perforator flap model was used according to our previous study (Qing et al., 2019). In short, the rats were anesthetized with pentobarbital sodium anesthesia (30 mg/kg, intraperitoneal). The flap was raised by sharp dissection in the plane between the panniculus carnosus and the deep fascia. On the dorsal side of each rat, an 11 cm × 3 cm multi-territory perforator flap area containing the iliolumbar vessel territory (area I), intercostal vessels territory (area II) and thoracodorsal vessels territory (area III) was designed. Then, iliolumbar vessels of the flap were used as a single vessel pedicle of multi-territory perforator flap by ligaturing the intercostal vessels and thoracodorsal vessels.

### 2.4 Drug Administration

The MLT group was injected with 40 mg/kg MLT (MedChemExpress, New Jersey, United States) intraperitoneally within 7 days after surgery (Gurlek et al., 2004; Venegas et al., 2012; Kerem et al., 2014). The control group was treated with an equal concentration of saline. Additionally, in the MLT and ML385 (MedChemExpress, New Jersey, United States) cotreatment group, the rats were injected with 40 mg/kg MLT and 30 mg/kg ML385 (Gou et al., 2020).

### 2.5 Flap Survival Evaluation

The flap was photographed and analyzed by digital imaging. We used Photoshop 7.0 software to assess the necrotic area of the flap 7 days post-operation. Flap necrosis is identified as dark areas or scarring of flap. Finally, the necrotic area was used to calculate the survival percentage of each flap area.

### 2.6 Infrared Detector

An infrared detector was used to analyze the blood flow temperature of the flaps in each group within 7 days after surgery. The area of the flap with a temperature less than 2°C was considered necrotic (Herrberger et al., 1989), and the survival percentage of each flap area was determined.

### 2.7 Histological Analysis

All the tissue samples were obtained from area II 7 days post-operation and then fixed with 4% paraformaldehyde for 1 day. The tissue was dehydrated and embedded in paraffin wax. Subsequently, the specimens were cut into 4 μm thick sections and stained with hematoxylin and eosin (H&E). Under a microscope (×40 magnification), we counted the microvessels of six arbitrary fields per section and calculated the mean vessel density (MVD) per unit area.

### 2.8 Immunohistochemistry

The skin flap from area II 7 days post-operation was fixed with 4% paraformaldehyde before embedding in paraffin wax. Six parts of each section were dewaxed in xylene, rehydrated through a graded set of ethanol baths, and then washed three times with PBS. Subsequently, the section was blocked with 3% hydrogen peroxide before repair in 10.2 mM sodium citrate buffer. After exposure to an antibody against CD31 (1:100, Affinity Bioscience, United States) overnight, followed by staining with PBS, the sections were exposed to an HRP-conjugated secondary antibody and counterstained with hematoxylin. We used Image-Pro Plus software to calculate absorption values that indicated CD31 expression, followed by immunohistochemistry (IHC) statistical calculation.

### 2.9 RT and Q-PCR

After surgery 7 days, we extracted the total RNA of the skin flap from area II using TRIzol reagent (Vazyme, Nanjing,China) and performed quantification using reverse transcription (RT) and polymerase chain reaction (PCR) with HiScript^®^ III RT SuperMix for qPCR (Vazyme, Nanjing,China). RT and qPCR were carried out with 1 µg of RNA and 4 µL of 4×gDNA wiper Mix remover in a total volume of 16 µL. The reactions were performed in a Thermo ProFlex PCR system (Applied Biosystems, United States) for 2 min at 42°C. Then, 4 µL 5×HiScript II qRT SuperMix II was transferred to a total volume of 20 µL. The reactions were similar to the former for 15 min at 50°C and 5 s at 85°C. The RT-PCR mix was diluted in nuclease-free water and stored at −20°C. Real-time PCR was performed using a LightCycler^®^ 480 II real-time PCR instrument (Roche, Switzerland) in a 20 μL PCR mixture containing 5 μL of cDNA, 10 μL of 2 × ChamQ universal SYBR qPCR Master Mix, 0.4 μL of forward primer, 0.4 μL of reverse primer, and 4.2 μL of nuclease-free water. The samples were incubated in a 96-well optical plate reader (Roche, Switzerland) at 95°C for 30 s, followed by 40 cycles of 95°C for 5 s and 60°C for 30 s. Each sample was assayed in triplicate. At the end of the qPCR program, melting curve analysis was performed to validate the specific generation of expected qPCR products. The primer sequences were designed in the laboratory and synthesized by TsingKe Biotech based on the mRNA sequences obtained from the NCBI database as follows: NRF2, 5′- GGA​GCA​ATT​CAA​CGA​AGC​TC-3′ (forward) and 5′-AAG​TTG​CCG​CTC​AGA​ACT​GT-3′ (reverse); FUN14 domain-containing protein 1 (FUNDC1), 5′-TGG​TGT​GCA​GGA​TTT​TTA​TTC​CA-3′ (forward) and 5′-AAC​TCT​CTT​CCA​GTC​GAT​CTG​T-3′ (reverse); VEGF, 5′-CAGAAGAACTT.

TTGGGCCGT-3′ (forward) and 5′-ACT​GTC​CTG​TGG​TGA​CTT​GT-3′ (reverse); and β-actin 5′-GCT​CGT​CGT​CGA​CAA​CGG​CTC-3′ (forward) and 5′-CAA​ACA​TGA​TCT​GGG​TCA​TCT​TCT​C-3′ (reverse). β-Actin expression was used as an internal control using the 2−ΔΔCt method to evaluate the expression level of the target gene.

### 2.10 Western Blotting

We extracted the protein from area II of every rat within 7 days after surgery and determined the protein concentration with the BCA method. Equivalent amounts of protein (30 µg per sample) were separated by gel electrophoresis and transferred to PVDF membranes. According to the molecular weight, the PVDF membranes were tailored to the target protein band. Next, the membranes were blocked in 5% skim milk at room temperature for 2 h and exposed to primary antibodies at 4°C overnight as follows: NRF2(1:1,000,Immunoway, California, United States),FUNDC1 (1:1,000, Immunoway, California, United States), VEGF (1:1,000, Immunoway, California, United States),BCL-2 (1:1,000, Immunoway, California, United States), Bax (1:1,000, Immunoway, California, United States),β-actin (1:5,000, Immunoway, California, United States),Caspase3 (1:2000,Abcam, Cambridge, United Kingdom) and α-tubulin (1:10,000,Abcam, Cambridge, United Kingdom). All antibodies are rabbit anti-rat IgG. Then, the membranes were washed with TBST and exposed to HRP-conjugated secondary antibodies (Affinity Bioscience, United States) for 2 h at room temperature. We used ECL to image bands with an imaging system (Bio–Rad, United States), and final protein quantification was performed with Image Lab (Bio–Rad, United States).

### 2.11 Immunofluorescence

Tissue of area II was fixed with 4% paraformaldehyde within 7 days after surgery, paraffin-embedded tissues were sectioned. Following three washes, the sections were incubated overnight with TUNEL (1:100, Invitrogen, Carlsbad, CA, United States) primary antibody at 4°C and then with secondary antibody for 1 h at room temperature. Subsequently, five areas of the stained sections were randomly selected for counting and observed with a Leica inverted microscope (Leica, Germany). The numbers of TUNEL-positive cell areas were analyzed by ImageJ software (Media Cybernetics).

### 2.12 Angiography

Five rats in each group underwent angiography 7 days post-operation. In summary, 5 g of gelatin was added to 100 ml of normal saline and dissolved at 40°C. Then, 80 mg of water-soluble red lead oxide was added to the former. The mixture was injected into the carotid artery of the rats until the limbs of the rats reddened. Next, the flaps were fixed for 24 h at 4°C and radiographed (55 kVp, 25 mA, 20 s exposure) with an X-ray machine (Fuji Computerized Radiography XG-1; Fujifilm, Tokyo, Japan).

### 2.13 Statistical Analysis

All statistical analyses were performed using SPSS 19.0 software (SPSS, IL, United States). The data are presented as the mean ± SEM. Statistically significant differences among the groups were assessed using Student’s *t* tests or one-way ANOVA, and *p* values <0.05 were considered statistically significant.

## 3 Results

### 3.1 Melatonin Promotes Skin Flap Survival

**FIGURE 1:**
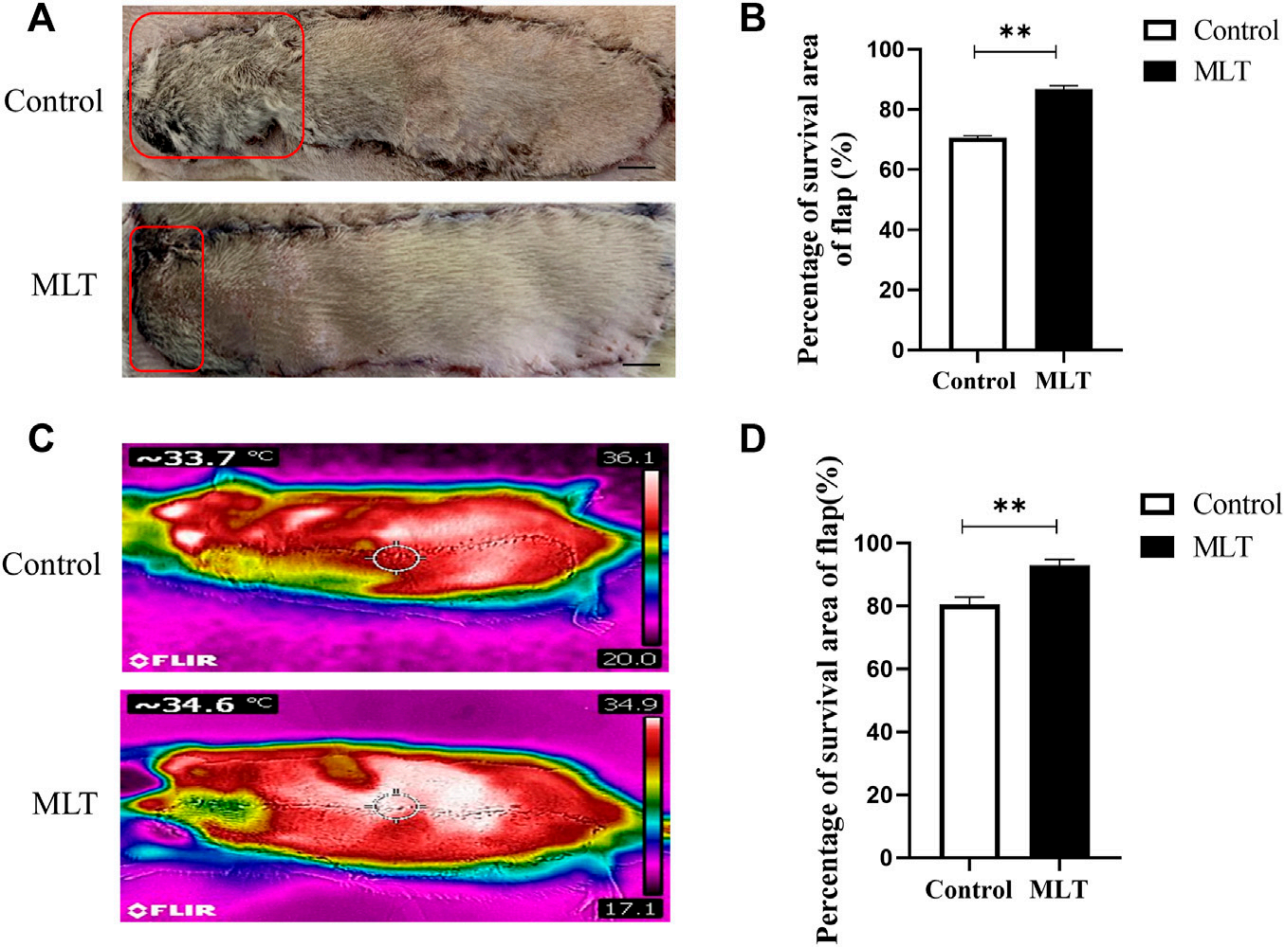
MLT improved multiterritory perforator flap survival and reduced multiterritory perforator flap necrosis. **(A)** Images of the skin flaps taken 7 days post-operation. The red pane is the area of flap necrosis we marked. Scale bar: 1 cm **(B)** Percentage of survival area of flap. Significant differences between the groups are indicated by **p < 0.001.**(C)** Images of the skin flaps taken by infrared detector. **(D)** Skin flap survival rate calculated by flap blood flow temperature. Values are the mean ± SEM, *n* = 6 per group. Significant differences between the groups are indicated by **p < 0.001. AbbreviationsMLT, melatonin.

All the rats survived the operation. The skin flap had a clear boundary between the normal and necrotic tissue, and the necrosis, which appeared dry and dark, was far from the area of the vessel pedicle. The percentages of skin flap survival between the control group and MLT group are shown in Figure 1A. The mean surviving areas were 85.334 ± 1.256% and 64.502 ± 1.232% in the MLT treatment and control groups, respectively (Figure 1B; *p* < 0.001). Moreover, infrared detection was used to analyze the blood flow temperature of the flaps in the two groups, and areas of the flaps with a temperature less than 2°C were considered necrotic. The flap survival area percentages were 92.843 ± 0.820% and 80.430 ± 0.960% in the MLT treatment and control groups, respectively (Figures 1C,D; *p* < 0.001). The results showed that the skin flap survival of the MLT group was higher than that of the control group. In other words, MLT promotes skin flap survival.

### 3.2 Melatonin Promotes Angiogenesis of Skin Flaps

**FIGURE 2:**
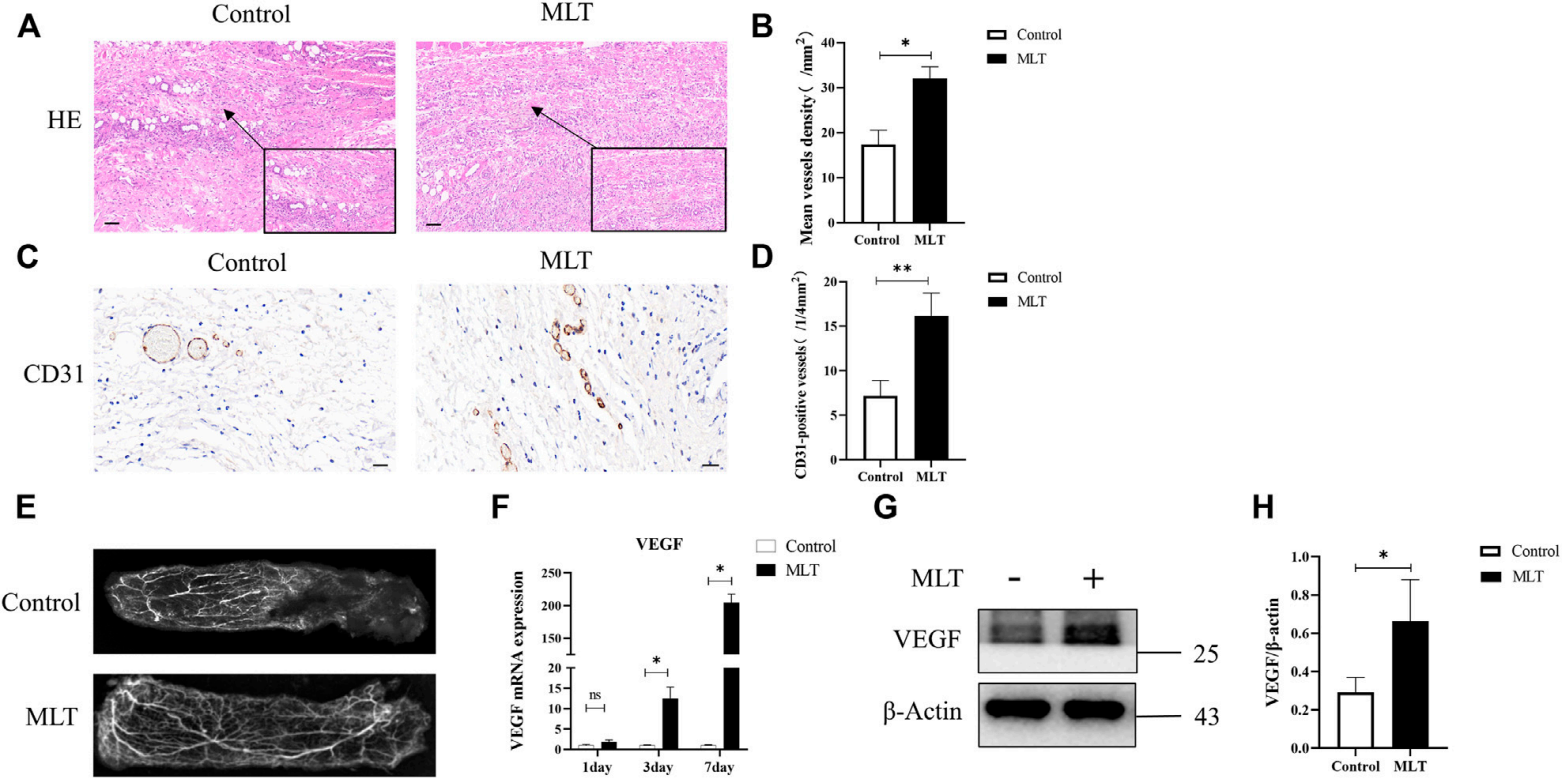
MLT promoted angiogenesis of skin flap. **(A)** H&E staining shows skin flap angiogenesis on postoperative day 7 (original magnification ×200). Scale bar: 50 μm. **(B)** The MVD per unit area assessed by H&E staining. Significant differences between the groups are indicated by *p < 0.05. **(C)** Immunochemical detection of CD31 (marker of angiogenesis) in the skin flap (original magnification ×400). Scale bar: 20 μm **(D)** The numbers of CD31-positive vessels. Values are the mean ± SEM, *n* = 6 per group. Significant differences between the groups are indicated by **p < 0.001. **(E)** Angiography showed areas I-III of the vasculature. The second choke zone II was significantly enhanced. **(F)** Expression of VEGF mRNA detected by qPCR. Significant differences between the groups are indicated by *p < 0.05.**(G)** Protein levels of VEGF and β-actin detected by Western blotting. **(H)** Quantitative analysis of the protein expression levels of VEGF and β-actin. Significant differences between the groups are indicated by *p < 0.05. Abbreviations: MLT, melatonin; MVD, mean vessel density.

To explore how MLT promotes skin flap survival, we carried out a histological examination. H&E staining revealed that angiogenesis was higher in the MLT group than in the control group. By randomly counting the vessel numbers of each group, the mean vessel densities of the two groups were 17.333 ± 3.180/mm^2^ and 32.000 ± 2.646/mm^2^ (Figures 2A,B; *p* < 0.05). CD31 is regarded as a marker of vessel endothelial cells, and immunohistochemical experiments were performed on tissue taken from area II. The results showed that the number of CD31-positive vessels in the MLT group was significantly higher than that in the control group. The numbers of CD31-positive vessels in the two groups were 16.167 ± 1.046/(1/4 mm^2^) and 7.167 ± 0.703 (1/4 mm^2^), respectively (Figures 2C,D; *p* < 0.001). In addition, angiograph revealed more choke vessels dilation in chokeII after MLT treatment. Additionally, vascular endothelial growth factor (VEGF) is considered to play the most important role in increasing angiogenesis and promoting vascular remodeling. Therefore, we determined the expression level of VEGF, and the results revealed that the expression of VEGF was upregulated both at the mRNA and protein levels (Figures 2F–H). The multi-territory flap was supplied by the single vascular pedicle, which supplied blood to the distal shaft type; therefore, the farther away from the pedicle, the less angiogenesis the flap had. Based on these data, we speculate that MLT promotes angiogenesis in the choke area.

### 3.3 Melatonin Inhibits Apoptosis During Flap Necrosis and Changes the Expression of Apoptosis-Related Proteins

**FIGURE 3:**
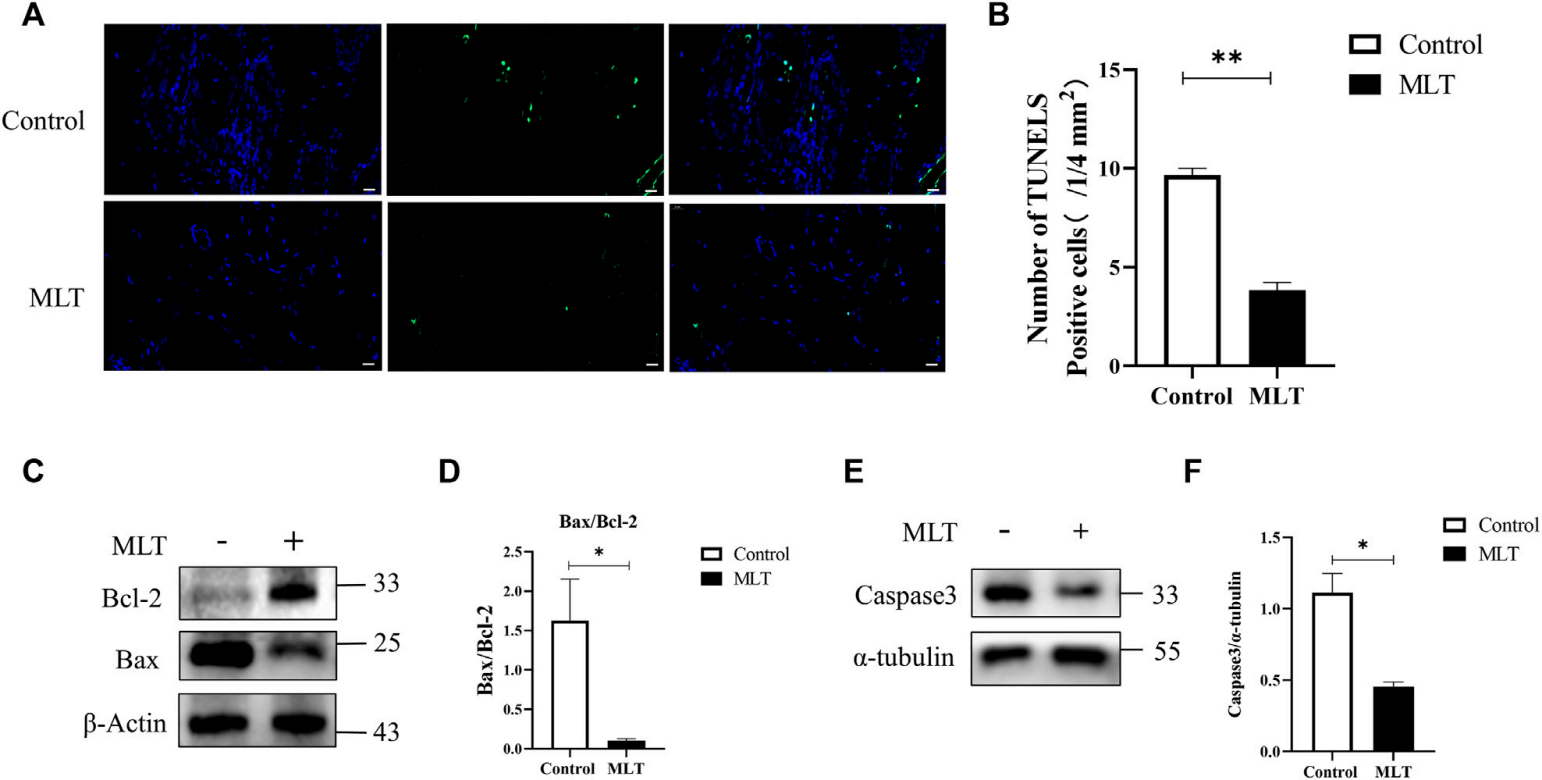
MLT suppressed apoptosis in the necrotic area of the flap. **(A)** Detection of apoptosis by TUNEL assay Scale bar: 20 μm. **(B)** Relative quantitative data of apoptotic cells and TUNEL cells. Significant differences between the groups are indicated by **p < 0.001. **(C)** Protein levels of Bcl-2, Bax, and β-actin detected by Western blotting. **(D)** Quantitative analysis of the protein expression levels of Bax/Bcl-2. Significant differences between columns are expressed as *p < 0.05. **(E)** Protein levels of caspase3 and α-tubulin detected by Western blotting. **(D)** Quantitative analysis of the protein expression levels of caspase3 and α-tubulin. Significant differences between columns are expressed as *p < 0.05. Abbreviations: MLT, melatonin.

To explore the effect of MLT on apoptosis, apoptotic cells were detected by TUNEL staining, the apoptosis-related proteins Bcl-2, Bax and apoptosis-initiating protein Caspase3 were assessed by western blotting. The results are shown in Figure 3A. The MLT group exhibited fewer TUNEL-positive cells, and the TUNEL-positive cells of the two groups were 9.667 ± 0.333 (1/4 mm^2^) and 3.833 ± 0.401 (1/4 mm^2^), respectively (Figure 3B). Western blotting analysis showed that the expression of the proapoptotic protein BCL-2 was upregulated while the antiapoptotic protein Bax was downregulated after MLT treatment (Figures 3C,D). In addition, expression of apoptosis-initiating protein Caspase3 was downregulated after MLT treatment (Figures 3E,F). Thus, we concluded that MLT could inhibit apoptosis to reduce flap necrosis and change the expression of apoptosis-related proteins.

### 3.4 Melatonin Alters the Expression Levels of NRF2 and FUNDC1

**FIGURE 4:**
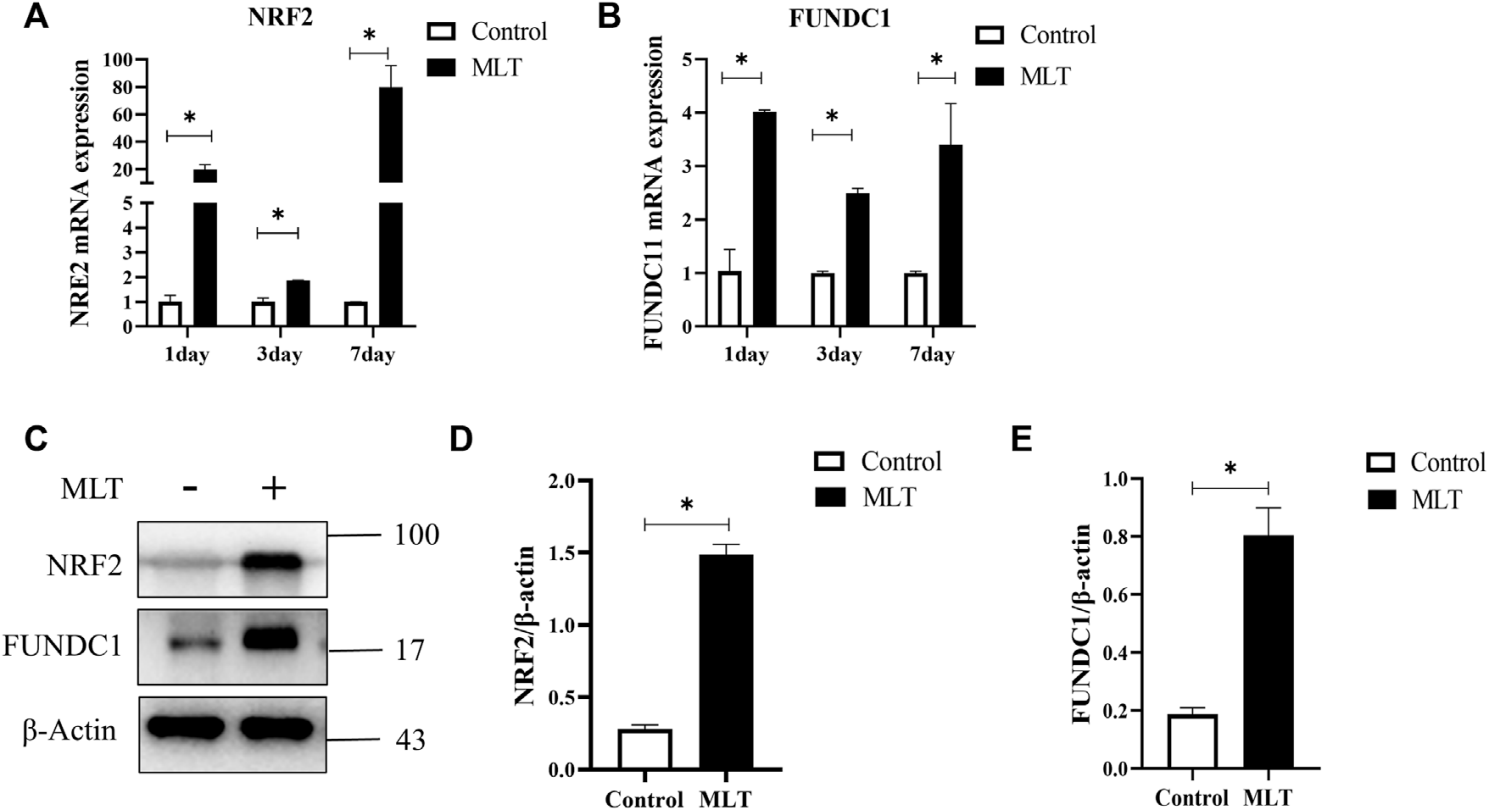
MLT regulated NRF2 and FUNDC1 gene expression. **(A)** Expression of NRF2 mRNA detected by qPCR. **(B)** Expression of FUDNC1 mRNA detected by qPCR. **(C)** Protein levels of NRF2, FUNDC1 and β-actin detected by Western blotting. **(D)** Quantitative evaluation of NRF2/β-actin. **(E)** Quantitative evaluation of FUNDC1/β-actin; the data are presented as the mean ± SEM, *p < 0.05 versus the control group, *n* = 3. Abbreviations: MLT, melatonin.

Based on a previous study (Fernández et al., 2015; Zhai et al., 2017; Ma Q. et al., 2020), we speculated that MTL could enhance the survival of multi-territory perforator flaps by inducing angiogenesis and suppressing apoptosis. However, further research is needed on the mechanism associated with actions of MLT. NRF2 and FUNDC1 are closely related to angiogenesis and apoptosis (Wang et al., 2018; Wang et al., 2019; Ma H. et al., 2020; Wang et al., 2021). By detecting the mRNA expression of NRF2 and FUNDC1, as shown in Figures 4A,B, the mRNA expression in the MLT group was higher than that in the control group on days 1, 3, and 7 post-operations. Additionally, protein expression of NRF2 and FUNDC1 significantly increased (Figures 4C–E). Those results suggested that MLT can promote the survival of multi-territory flaps and change the correlation factor levels of NRF2 and FUNDC1.

### 3.5 Melatonin Improves the Survival of Multi-Territory Perforator Flaps by Inducing Angiogenesis and Suppressing Apoptosis via NRF2 Activation

**FIGURE 5:**
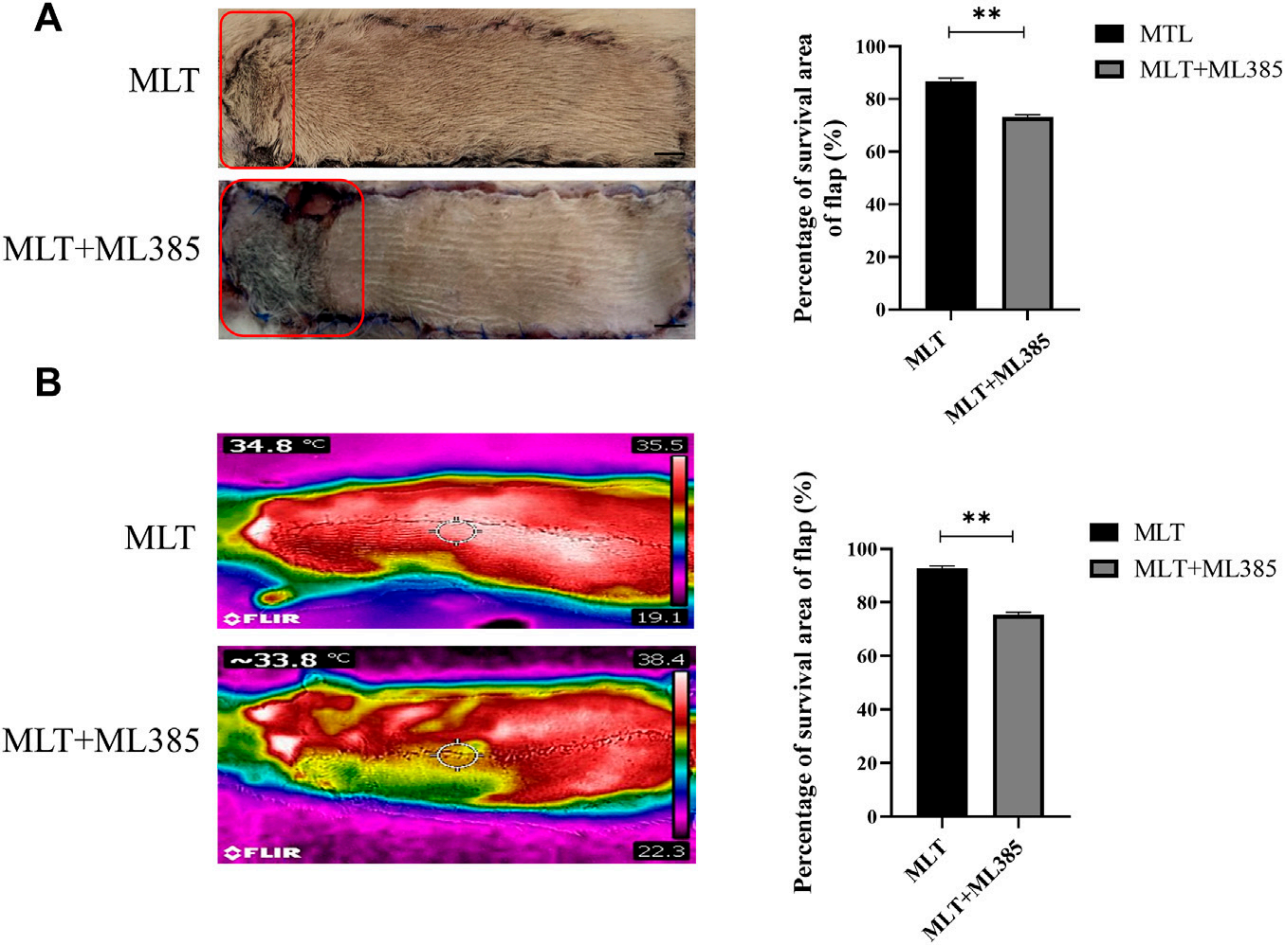
MLT activated NRF2 to increase the survival area of the multidomain perforator flap. **(A)** Flap necrosis in the MLT group and MLT + ML386 group on postoperative day 7 and relative quantification of the flap survival rate. The red pane is the area of flap necrosis we marked. Significant differences between groups are expressed as **p < 0.001. Scale bar: 1 cm **(B)** Flap blood flow temperature in the MLT group and MLT + ML386 group and quantitative estimation of the flap survival rate. Columns represent the mean ± SEM, **p < 0.001. Abbreviations: MLT, melatonin.

**FIGURE 6:**
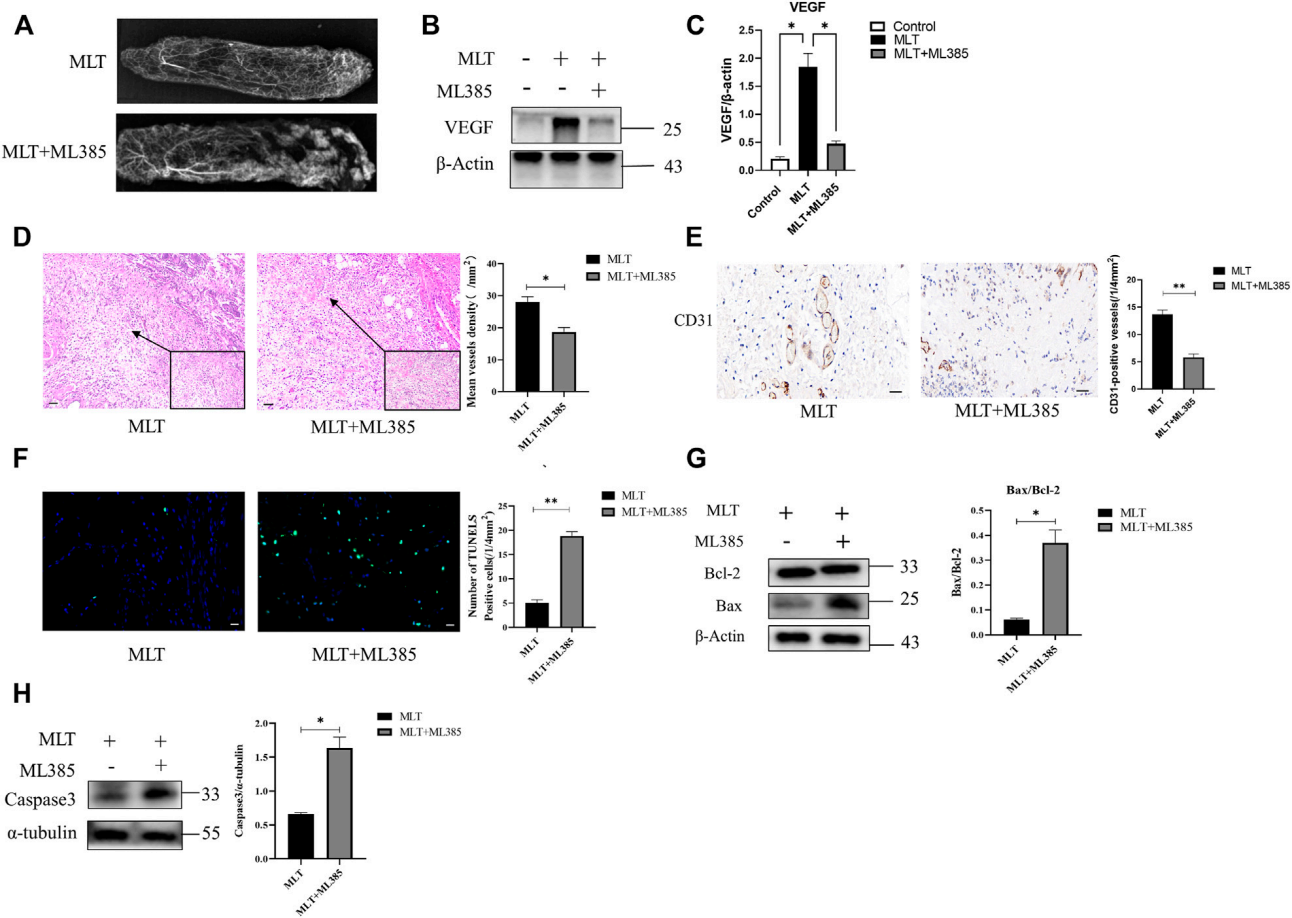
MLT promoted angiogenesis and inhibited apoptosis in flaps by stimulating NRF2. **(A)** Angiography showed obvious angiogenesis in the vascular territory of areas I-III. **(B)** Expression level of VEGF protein. **(C)** Expression of VEGF mRNA. *p < 0.05 versus the MLT group. **(D)** Tissue sections after H&E staining and relative quantification of MVD. Differences between groups are expressed as *p < 0.05. Scale bar: 50 μm **(E)** Expression of CD31 by immunohistochemistry. Values are the mean ± SEM, *n* = 6 per group. Significant differences between the groups are indicated by**p < 0.001. Scale bar: 20 μm **(F)** Immunofluorescence detection of apoptotic cells and TUNEL cells in two groups; Column mean ± SEM, *n* = 6, compared with MLT group **p < 0.001. Scale bar: 20 μm **(G)** The expression and quantitative analysis of the apoptosis-related proteins Bax and Bcl-2 by Western blotting. Columns represent the mean ± SEM, *p < 0.05 versus the MLT group. **(H)** The expression and quantitative analysis of caspase3 and α-tubulin proteins by Western blotting. Columns represent the mean ± SEM, *p < 0.05 versus the MLT group.Abbreviations: MLT, melatonin; MVD, mean vessel density.

Hence, to explore the mechanism by which MLT mediates protection via NRF2, the rats were divided into three groups: the control group, MLT group, and MLT + ML385 cotreatment group. As shown in Figure 5A, inhibition of NRF2 activity reversed the positive effect of MLT on the area of flap survival. The mean surviving areas were 86.867 ± 1.102% and 73.217 ± 0.789% in the MLT treatment group and the MLT + ML385 cotreatment group, respectively (Figure 5B; *p* < 0.001). The percent area of flap survival estimated by infrared detectors in the two groups was 92.843 ± 0.802% and 75.367 ± 0.974%, respectively (Figures 5C,D; *p* < 0.001). Thus, it was concluded that MLT mediated the promotion of flap survival through the activation of NRF2. In addition, angiography revealed that NRF2 inhibitor reversed the dilation of choke vessels at the distal end of the flap, and the protein expression of VEGF was also reduced, indicating that MLT could promote flap angiogenesis and dilate choke vessels by activating NRF2 (Figures 6A–C; *p* < 0.001). Similarly, inhibition of NRF2 reduced the MVD and CD31-positive vessels, as shown by H&E staining and immunohistochemistry, respectively (Figure 6D; *p* < 0.05 and Figure 6E; *p* < 0.001). These results illustrated that MLT promotes angiogenesis in the areaⅡ of the skin flap and dilates choke vessels of choke II by regulating NRF2. In addition, inhibition of NRF2 could reverse the effect of MLT in suppressing apoptosis and increase caspase3 protein expression and Bax/Bcl-2 protein ratio, which illustrated that MLT could suppress apoptosis by regulating NRF2 (Figure 6F; *p* < 0.001 and Figures 6G,H; *p* < 0.05). These results reveal that MLT activates NRF2 to promote angiogenesis and inhibit apoptosis in skin flaps.

### 3.6 Melatonin Affects Skin Flaps via the NRF2/FUNDC1 Axis

**FIGURE 7:**
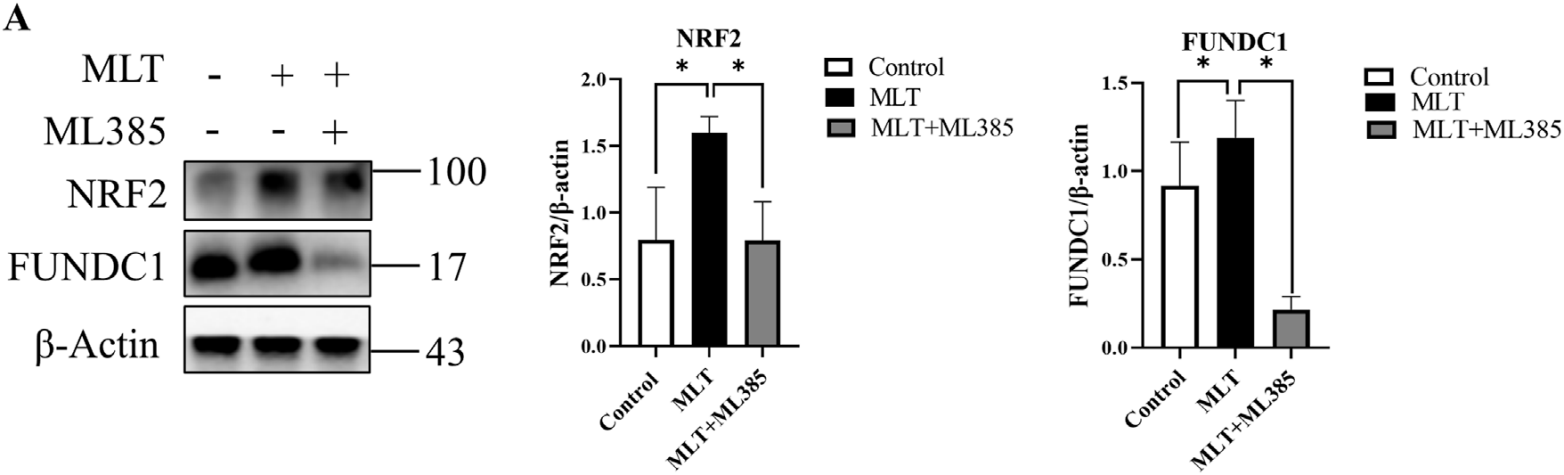
MLT functions through the NRF2/FUNDC1 axis. **(A)** The mRNA and protein expression levels of NRF2 and FUNDC1 were detected by qPCR and Western blotting, respectively; the expression of NRF2 and FUNDC1 was estimated relative to that of β-actin. *p < 0.05 versus the MLT group.

As mentioned above, the expression levels of NRF2 and FUNDC1 were upregulated after MLT treatment; however, their relationship was not yet known. By inhibiting the expression of NRF2, we found that the effect of melatonin on promoting flap survival was reversed. In addition, the expression level of FUNDC1 was also reduced (Figure 7). Therefore, we reasonably speculated that the NRF2/FUNDC1 axis might mediate the influence of MLT on perforator flap survival.

## 4 Discussion

MLT is an active hormone secreted by the conarium and has antioxidant stress and anti-inflammatory functions (Reiter et al., 2014; Reiter et al., 2017; Amaral and Cipolla-Neto 2018; Cipolla-Neto and Amaral 2018; Hardeland 2018). Based on previous research, MLT protects against ischemia-reperfusion and cancer, which are closely related to improving angiogenesis and suppressing apoptosis (Yeung et al., 2015; Sun et al., 2016; Gou et al., 2020; Mehrzadi et al., 2021). However, it is unclear what effect MLT has on multi-territory perforator flaps. Thus, in this study, we attempted to explore the effect of MLT on multi-territory perforator flaps and its possible molecular mechanism.

With the in-depth study of multi-territory perforator flaps, remodeling of choke vessels is considered to play a significantly important role in promoting multi-territory perforator flaps (Taylor et al., 2013). Under physiological conditions, the choke area is generally closed, and when the flap is too large to exceed the area supplied by a single angiosome, the survival of the multi-territory perforator flap depends on the transformation of the choke area to a true anastomosis (Zhuang et al., 2012; Fang et al., 2020). Therefore, we constructed a multi-territory perforator flap for related research and selected the tissue from the choke area for analysis. First, we found that compared to wild-type controls, MLT-treated rats had a reduced area of ischemic necrosis of the skin flap. Then, histological analysis comparing the MVD and CD31-positive vessels between the MLT group and the control group revealed that MLT increased angiogenesis in the choke area. Following angiography, the author found that dilation was significantly increased in choke zone II in the MLT-treated group, while there was almost no dilation in control group. Thus, we concluded that MLT can promote the survival of multi-territory perforator flaps through remodeling of choke vessels.

Apoptosis is programmed cell death and an important way to maintain homeostasis. Studies (Hosseinzadeh et al., 2016; Zhai et al., 2017) have shown that MLT has a protective effect on osteoarthritis and myocardial ischemia reperfusion injury by inhibiting apoptosis. The regulation of apoptosis could significantly reduce the rate of flap necrosis (Mao et al., 2019). Bcl-2 and Bax are two key proteins important in regulating cell apoptosis. Among them, Bcl-2 has an anti-apoptotic effect, while Bax has the opposite effect. In addition, activation of Caspase3 was considered an important step in initiating the apoptosis program (Cohen 1997). Western blotting analysis showed that the Bax/Bcl-2 ratio and Caspase3 in the MLT treatment group was significantly lower than that in the control group, which illustrated that MLT inhibited the apoptosis signaling pathways. Then, we used immunofluorescence to explore the effect of MLT on apoptosis. The results showed that the MLT-treated group had fewer apoptotic cells, which illustrated that MLT has a protective effect on multi-territory perforator flaps by inhibiting apoptosis.

**FIGURE 8:**
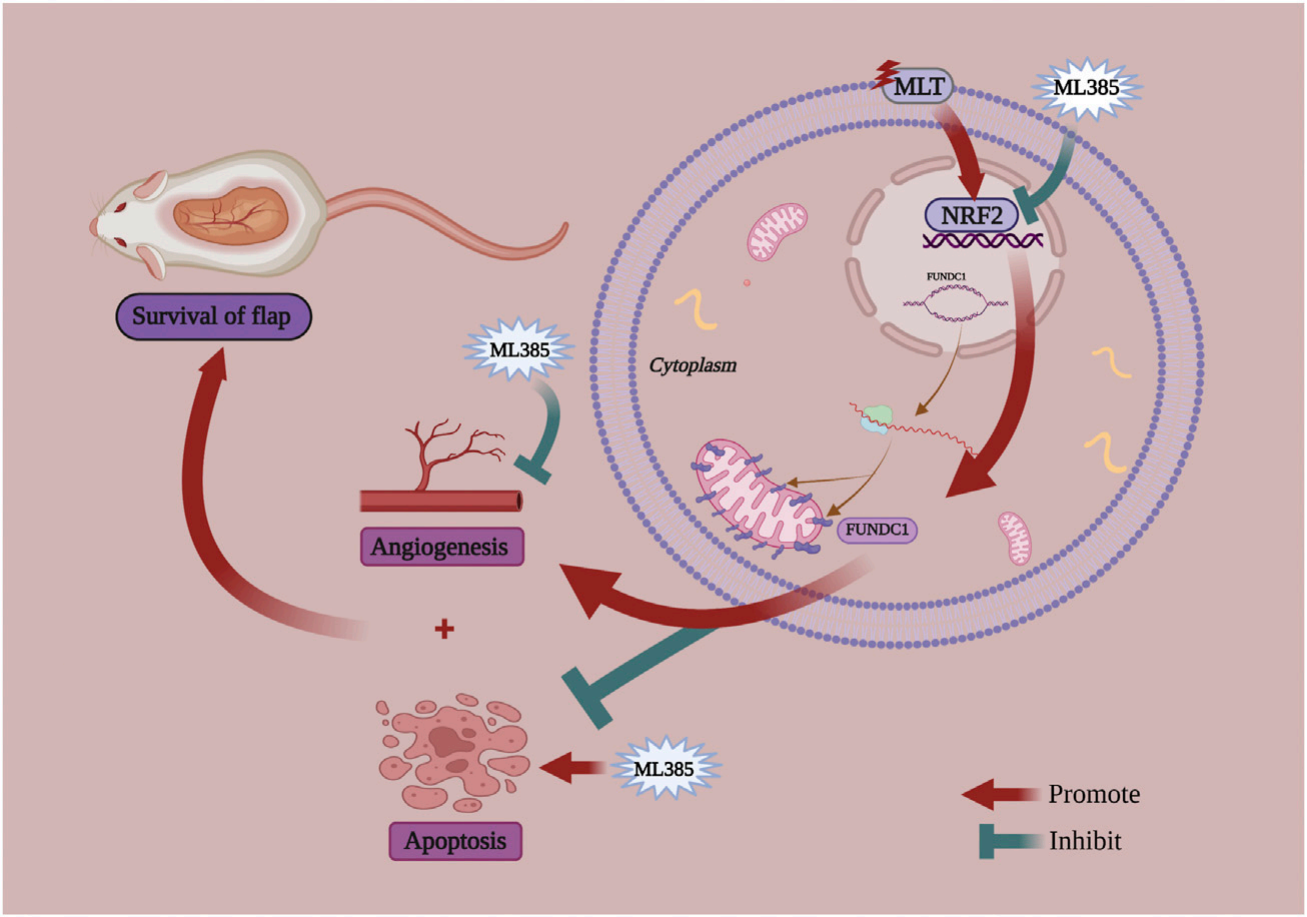
A schematic diagram illustrating MTL improve the survival of multiterritory perforator flaps by promoting angiogenesis and inhibiting apoptosis via the NRF2/FUNDC1 axis.

At present, there are two theories regarding the factors affecting choke vessel remodeling: blood flow stress shearing or hypoxia-mediated vascular endothelial cell migration and proliferation (Hudlicka and Brown 2009; Qing et al., 2017). Previous studies (Kahroba and Davatgaran-Taghipour 2020; Lin et al., 2020) have shown that the stress response of the vascular endothelium resists various pathological damages, such as blood flow shear stress and oxidative stress. NRF2 is the most important transcriptional regulator in the regulation of the stress response and maintains the homeostasis of vascular endothelial cells. NRF2 has been demonstrated to play an important role in angiogenesis and can upregulate the expression of VEGF, which is the most important factor in vascular remodeling (Pendyala et al., 2011; Kuang et al., 2013; Ji et al., 2014). Researchers have found that MLT can activate NRF2 to suppress apoptosis (Wang et al., 2019; Ma H. et al., 2020). Chang et al. (Chang et al., 2018) also proposed that MLT has a protective effect against apoptosis via the NRF2 signaling pathway. Interestingly, Wang et al. (Wang et al., 2021) proposed that FUNDC1 was an integral mitochondrial outer-membrane protein that could increase the expression of VEGF and promote endothelial cell angiogenesis. Similarly, FUNDC1 not only alleviates ischemia–reperfusion injury but also inhibits apoptosis (Wang et al., 2018; Jiang X. et al., 2021; Cai et al., 2021; Li et al., 2021). In our study, we observed that MLT treatment reduced the area of ischemic necrosis of the flap. Following analysis of the flap tissue, we found that angiogenesis in the choke area was increased, while apoptosis was decreased. These results confirmed that MLT could reduce the ischemic necrosis of the multi-territory perforator flap by promoting angiogenesis and suppressing apoptosis. However, the molecular mechanism that promotes the survival of flaps is unclear. In our study, real-time PCR was used to detect gene expression and indicated that the mRNA expression of NRF2 and FUNDC1 was upregulated after MLT treatment. Following western blot analysis, the results also showed increased protein expression of NRF2 and FUNDC1. Thus, MLT could indeed upregulate the expression levels of NRF2 and FUNDC1, but their relationship has not yet been determined. To verify the association between NRF2 and FUNDC1, we used cotreatment with MLT and the NRF2 inhibitor ML385. After MLT and ML385 cotreatment, the results showed that the effect of MLT in promoting multi-territory perforator flaps by inhibiting apoptosis and promoting angiogenesis was reversed. The expression of FUNDC1 protein was downregulated. Therefore, we speculated that MLT promotes angiogenesis and suppresses apoptosis via the NRF2/FUNDC1 axis, thereby promoting the survival of multi-territory perforator flaps (Figure 8).

Perforator flaps have become a common method for repairing large soft tissue defects. Among them, conjoined perforator flaps, combined perforator flaps, and multi-territory perforator flaps are all reconstruction methods that could solve those clinical problem. Conjoined flap supplied by two adjacent angiosomes to expand the incision area of the flap by applying technology of supercharged or tubercharged (Hallock 2008), which have the disadvantage of being difficult to harvest. Combined flap is a combination of two different skin flaps, which has the disadvantage of sacrificing the second donor site (He et al., 2021). While multi-territory perforator flap has the advantages of simple incision and large acquisition area. However, the unreliable blood supply of multi-territory perforator flaps restricts the extensive clinical application of multi-territory perforator flaps. As a result, Promoting the survival area of multi-territory perforator flaps has important clinical significance.

In addition, there are exist certain limitation that therapy of melatonin applies to multi-territory perforator flaps. For example, further large animal models and clinical trials need be explored for the safety of melatonin, and the side effects of the drug need to be considered. In addition, the effect of melatonin on the human body needs and to be verified in the future.

In summary, this study investigated the effect of MLT on the survival area of multi-territory perforator flaps. The results showed that MLT could increase the survival area of multi-territory perforator flaps by promoting angiogenesis and inhibiting apoptosis. This process may be related to NRF2/FUNDC1 axis.

## Data Availability

The raw data supporting the conclusions of this article will be made available by the authors, without undue reservation.
